# Supplementary material for: Sedentary Behaviour and Physical Activity Levels during Second Period of Lockdown in Chilean’s Schoolchildren: How Bad Is It?
Source: Children (Basel). 2023 Mar 1;10(3):481. doi: 10.3390/children10030481 (PMC10047652; doi:10.3390/children10030481)
Supplement: Supplementary file 1 [file children-10-00481-s001.zip › children-2199811-supplementary.pdf]

PONTIFICIA UNIVERSIDAD CATÓLICA DE VALPARAÍSO

CUESTIONARIO PADRES E HIJOS ACTIVOS Y SALUDABLES (EPAS/ EHAS)

ASENTIMIENTO INFORMADO

"Entorno familiar y escolar. Efectos de los niveles de actividad física de los padres sobre la actividad física de los niños".

Somos un grupo de investigación formado por profesores de la Pontificia Universidad Católica de Valparaíso, Chile y la Universidad de Cádiz, España que trabajamos en actividad física y salud. Estamos realizando un estudio conforme a las necesidades educativas y de actividad física de nuestros escolares. Este estudio tiene como fin, indagar sobre el entorno **familiar y escolar**, y la asociación de los niveles de actividad física de los padres sobre la actividad física de sus hijos. La información obtenida, nos permitirá identificar factores fundamentales para mejorar métodos de enseñanza en prácticas físicas y aumentar los niveles de actividad física en la población infantil y juvenil. Para ello, necesitamos que los/as participantes contesten a este cuestionario. Al mismo tiempo necesitamos que uno de sus hijos en etapa escolar pueda contestar el **cuestionario de escolares** adjunto a esta solicitud.

Usted debe saber que:

a. Su participación en este estudio es totalmente **voluntaria.**

b. La participación o no participación no supondrá para usted **ningún riesgo.**

c. Todos los datos obtenidos en este estudio serán confidenciales y se tratarán conforme establece la Ley Orgánica de Protección de Datos Personales y garantía de derechos digitales 3/2018 de España y la Ley N.º 19628 sobre Protección de la Vida Privada en Chile.

d. La información obtenida únicamente se utilizará para los fines específicos de investigación.

e. Para cualquier duda o consulta puede escribir al coordinador del estudio, profesor Fernando Rodríguez al mail **fernando.rodriguez@pucv.cl.**

6. Este estudio está patrocinado por la **Escuela de Educación Física** y financiado por la **Dirección de Investigación de la Pontificia Universidad Católica de Valparaíso.**

¿Acepta participar en este estudio?

____

NOMBRE Y APELLIDO

INFORMACIÓN PERSONAL (HIJO/A)

1. Nombre y apellidos ____________________________________ **2. Fecha de nacimiento** __/__/___

3.Edad __________________ **4.Sexo** **Hombre** ☐ **Mujer** ☐

5. Dirección en la cual resides (ciudad, calle, número, departamento, letra) _________________________________

6.Nombre de tu establecimiento educacional ____

7. ¿En qué curso vas? __________________

8. ¿Tu familia posee algún automóvil, furgoneta o camioneta?

No ☐ Sí, uno ☐ Sí, dos o más ☐

9. ¿Tienes tu propia habitación? Sí ☐ No ☐

10. Durante los últimos 12 meses, ¿cuántas veces viajaste de vacaciones con tu familia?

Ninguna ☐ Una ☐ Dos ☐ Más de dos ☐

11. ¿Cuántos computadores tiene tu familia en casa?

Ninguno ☐ Uno ☐ Dos ☐ Más de dos ☐

MODO DE DESPLAZAMIENTO

12. ¿Cuánta distancia te desplazas al colegio?

	**No me desplazo al colegio**	**Menos de 0,5 km (menos de 5 cuadras)**	**Entre 0,5 a 1 km (Entre 5 y 10 cuadras)**	**1 a 2 km (10 a 20 cuadras)**	**2 a 3 km**	**3 a 5 km**	**Más de 5 km**
Antes **de la pandemia**	☐	☐	☐	☐	☐	☐	☐
Durante **la pandemia**	☐	☐	☐	☐	☐	☐	☐

13. ¿Cuánto tardas en llegar al colegio desde que sales de tu casa?

	**No me desplazo al colegio**	**Menos de 15 min**	**Entre 15 a 30 min**	**Desde 30 a 60 min**	**Más de 60 min**
Antes **de la pandemia**	☐	☐	☐	☐	☐
Durante **la pandemia**	☐	☐	☐	☐	☐

14. ¿Cómo VAS habitualmente al colegio?

	**No me desplazo al colegio**	**Caminando**	**Bicicleta**	**Automóvil/colectivo/taxi/Uber**	**Moto**	**Autobús (micro)**	**Tren/Metro**	**Otra**
Antes **de la pandemia**	☐	☐	☐	☐	☐	☐	☐	☐
Durante **la pandemia**	☐	☐	☐	☐	☐	☐	☐	☐

15. ¿Cómo VUELVES del colegio a casa?

	No me desplazo al colegio	Caminando	Bicicleta	Automóvil/colectivo/taxi/Uber	Moto	Autobús (micro)	Tren/Metro	Otra
Antes **de la pandemia**	☐	☐	☐	☐	☐	☐	☐	☐
Durante **la pandemia**	☐	☐	☐	☐	☐	☐	☐	☐

16. ¿Con quién VAS al colegio?

	No me desplazo al colegio	Con mi padre	Con mi madre	Con mis amigos	Con alguno de mis abuelos	Yo solo o sola	Con vecinos	Con mi hermano/a
Antes **de la pandemia**	☐	☐	☐	☐	☐	☐	☐	☐
Durante **la pandemia**	☐	☐	☐	☐	☐	☐	☐	☐

17. ¿Con quién VUELVES del colegio?

	No me desplazo al colegio	Con mi padre	Con mi madre	Con mis amigos	Con alguno de mis abuelos	Yo solo o sola	Con vecinos	Con mi hermano/a
Antes **de la pandemia**	☐	☐	☐	☐	☐	☐	☐	☐
Durante **la pandemia**	☐	☐	☐	☐	☐	☐	☐	☐

PERFIL DE ACTIVIDAD FÍSICA EN JÓVENES (YAP)

18. ¿Cuántos días a la semana tienes clases de Educación Física?

	Cero días (nunca)	Un día	Dos días	Tres días	Cuatro días	Cinco días
Antes **de la pandemia**	☐	☐	☐	☐	☐	☐
Ahora durante **la pandemia**	☐	☐	☐	☐	☐	☐

19. ¿Cuántos recreos tienes al día?

	Cero (ninguno)	Un recreo	Dos recreos	Tres recreos	Cuatro recreos
Antes **de la pandemia**	☐	☐	☐	☐	☐
Ahora durante **la pandemia**	☐	☐	☐	☐	☐

20. En general, ¿disfrutas realizar actividad física?

	Nada	Poco	Algo	Bastante	Mucho
Antes **de la pandemia**	☐	☐	☐	☐	☐
Ahora durante **la pandemia**	☐	☐	☐	☐	☐

21. ¿Disfrutas en las clases de Educación Física?

	Nada	Poco	Algo	Bastante	Mucho
Antes **de la pandemia**	☐	☐	☐	☐	☐
Ahora durante **la pandemia**	☐	☐	☐	☐	☐

Ir al colegio

22. ¿Cuántos días FUISTE caminando o en bicicleta al colegio? (si no lo recuerdas con exactitud, intenta señalar la respuesta más adecuada)

	Cero días (nunca)	Un día	Dos días	Tres días	Cuatro días	Cinco días
Antes de la pandemia	☐	☐	☐	☐	☐	☐
Ahora durante la pandemia	☐	☐	☐	☐	☐	☐

Actividad durante las clases de Educación Física

23. Durante las clases de Educación Física, ¿con qué frecuencia estuviste corriendo y moviéndote en juegos o actividades organizadas por el profesor? (si no tuviste Educación Física, elige "no tuve Educación Física")

	No tuve educación física	Muy poco tiempo	Poco tiempo	Más o menos la mitad del tiempo	Mucho tiempo	Casi todo el tiempo
Antes de la pandemia	☐	☐	☐	☐	☐	☐
Ahora durante la pandemia	☐	☐	☐	☐	☐	☐

Actividad durante los recreos

24. Durante los recreos, ¿con qué frecuencia estuviste practicando deporte, caminando, corriendo o jugando de forma activa? (si no tuviste recreos, elige "no tuve recreos en el colegio")

	No tuve educación física	Muy poco tiempo	Poco tiempo	Más o menos la mitad del tiempo	Mucho tiempo	Casi todo el tiempo
Antes de la pandemia	☐	☐	☐	☐	☐	☐
Ahora durante la pandemia	☐	☐	☐	☐	☐	☐

Actividad durante el descanso para comer

25.Durante el descanso para almorzar ¿con qué frecuencia estuviste moviéndote, caminando o jugando? (si no tienes descansos para comer en el colegio porque no hay clases por la tarde, elige "no tuve descanso para comer")

	No tuve educación física	Muy poco tiempo	Poco tiempo	Más o menos la mitad del tiempo	Mucho tiempo	Casi todo el tiempo
Antes de la pandemia	☐	☐	☐	☐	☐	☐
Ahora durante la pandemia	☐	☐	☐	☐	☐	☐

FUISTE del colegio

26. ¿Cuántos días volviste del colegio caminando o en bicicleta? (si no lo recuerdas con exactitud, intenta señalar la respuesta más adecuada)

	Cero días (nunca)	Un día	Dos días	Tres días	Cuatro días	Cinco días
Antes de la pandemia	☐	☐	☐	☐	☐	☐
Ahora durante la pandemia	☐	☐	☐	☐	☐	☐

Volver del colegio

27. ¿Cuántos días volviste del colegio caminando o en bicicleta? (si no lo recuerdas con exactitud, intenta señalar la respuesta más adecuada)

	Cero días (nunca)	Un día	Dos días	Tres días	Cuatro días	Cinco días
Antes de la pandemia	☐	☐	☐	☐	☐	☐
Ahora durante la pandemia	☐	☐	☐	☐	☐	☐

Estas preguntas son sobre tu nivel de actividad física en distintos periodos (fuera del colegio). Responde pensando en la actividad física que has hecho fuera del colegio durante los últimos siete días.

Actividad ANTES DEL COLEGIO

28. Antes de empezar el colegio (entre las 6:00 y las 8:00 de la mañana), ¿cuántos días hiciste actividad física durante al menos 10 minutos? (aquí se incluyen actividades realizadas en casa, colegio u otro lugar, pero NO incluye el ir caminando o en bicicleta al colegio)

	Cero días (nunca)	Un día	Dos días	Tres días	Cuatro días	Cinco días
Antes **de la pandemia**	☐	☐	☐	☐	☐	☐
Ahora durante **la pandemia**	☐	☐	☐	☐	☐	☐

Actividad POR LAS TARDES

29. Por las tardes (entre las 18:00 y las 22:00), ¿cuántos días hiciste actividad física durante al menos 10 minutos? (se incluyen actividades como jugar con amigos/familia, deportes de equipo o clases en las que hagas actividad física (como danza, natación u otras), pero NO la vuelta del colegio caminando o en bicicleta)

	Cero días (nunca)	Un día	Dos días	Tres días	Cuatro días	Cinco días
Antes **de la pandemia**	☐	☐	☐	☐	☐	☐
Ahora durante **la pandemia**	☐	☐	☐	☐	☐	☐

Actividad en FIN DE SEMANA (sábado y domingo).

30. ¿Cuánta actividad física hiciste el sábado pasado? (se incluye ejercicio físico, tareas de la casa, excursiones (paseos) con la familia, deportes, baile o juegos. Si no lo recuerdas con exactitud, intenta señalar la respuesta más adecuada)

	Nada de actividad física (0 minutos)	Muy poca actividad física (1-30 minutos)	Una cantidad media de actividad física (31 – 59 minutos)	Mucha actividad física (1-2 horas)	Una gran cantidad de actividad física (más de dos horas)
Antes **de la pandemia**	☐	☐	☐	☐	☐
Ahora durante **la pandemia**	☐	☐	☐	☐	☐

TIEMPO SEDENTARIO

Estas preguntas son sobre el tiempo que pasas descansando y sentado. Probablemente estés sentado mientras comes, haces las tareas (deberes) o tocas instrumentos musicales; pero también puedes estar sentado cuando ves la televisión, usas videojuegos, usas el computador (ordenador, portátil) el teléfono móvil (celular), tablets/iPad u otros. Responde a estas preguntas pensando en el tiempo que has pasado sin moverte durante estas actividades en los últimos sietedías (solo en los días de colegio)

Tiempo viendo TELEVISIÓN

31. ¿Cuánto tiempo estuviste viendo la televisión fuera del colegio? (incluye el tiempo que estuviste viendo películas (dibujos animados) o deportes, pero NO jugando a videojuegos)

	No vi nada de televisión	Vi la televisión menos de 1 hora al día	Vi la televisión entre 1- 2 horas al día	Vi la televisión más de 2 horas y hasta 3 horas al día	Vi la televisión más de 3 horas al día
Antes **de la pandemia**	☐	☐	☐	☐	☐
Ahora durante **la pandemia**	☐	☐	☐	☐	☐

Tiempo con VIDEOJUEGOS

32. ¿Cuánto tiempo estuviste jugando a videojuegos fuera del colegio? (incluye jugar a la Nintendo DS, Wii, Xbox, PlayStation, juegos en tu teléfono celular, tablets/iPad u otras consolas). NO incluir juegos con computador

	No jugué nada con consolas	Jugué menos de 1 hora al día	Jugué entre 1-2 horas al día	Jugué más de 2 horas y hasta 3 horas al día	Jugué más de 3 horas al día
Antes **de la pandemia**	☐	☐	☐	☐	☐
Ahora durante **la pandemia**	☐	☐	☐	☐	☐

Tiempo con COMPUTADOR

33. ¿Cuánto tiempo estuviste usando el computador fuera del colegio? (NO se incluye el uso para hacer tareas, pero SÍ el tiempo en Facebook, usando internet, chateando, jugando a videojuegos o juegos online)

	No usé el computador para estas actividades	Usé el computador menos de 1 hora al día	Usé el computador entre 1 -2 horas al día	Usé el computador más de 2 horas y hasta 3 horas al día	Usé el computador más de 3 horas al día
Antes **de la pandemia**	☐	☐	☐	☐	☐
Ahora durante **la pandemia**	☐	☐	☐	☐	☐

Tiempo con TELÉFONO MÓVIL

34. ¿Cuánto tiempo estuviste usando tu teléfono móvil (celular) fuera del colegio? (esto incluye el tiempo hablando por teléfono y escribiendo mensajes). Si no tienes teléfono y no usas nunca el de tus padres o algún amigo, elige la opción "no usé nunca el teléfono móvil".

	No usé el teléfono móvil	Usé el teléfono móvil menos de 1 hora al día	Usé el teléfono entre 1-2 horas al día	Usé el teléfono móvil más de 2 horas y hasta 3 horas al día	Usé el teléfono móvil más de 3 horas al día
Antes **de la pandemia**	☐	☐	☐	☐	☐
Ahora durante **la pandemia**	☐	☐	☐	☐	☐

35. Hábitos sedentarios en una semana normal (NO solo la última semana): ¿Cuál de las siguientes frases define mejor tus hábitos sedentarios en casa?

	Apenas estoy sentado en mi tiempo libre	Estoy sentado durante poco tiempo en mi tiempo libre	Estoy sentado mucho tiempo en mi tiempo libre	Estoy sentado mucho tiempo en mi tiempo libre	Estoy sentado casi todo el tiempo en mi tiempo libre
Antes **de la pandemia**	☐	☐	☐	☐	☐
Ahora durante **la pandemia**	☐	☐	☐	☐	☐

CUESTIONARIO DE AUTOEVALUACIÓN DE LA CONDICIÓN FÍSICA Y LA CALIDAD DE VIDA

Es muy importante que conteste a las siguientes preguntas usted solo, sin tener en cuenta las respuestas de otras personas. Sus respuestas solo son útiles para el progreso de la ciencia. Por favor, conteste todas las preguntas con sinceridad y no las deje en blanco. Y aún más importante, sea sincero. Gracias por su cooperación con la ciencia.

Piense sobre su nivel de condición física (comparado con sus amigos/as) y elija la opción más adecuada para cada pregunta.

36. Mi condición física general es:

	Muy mala	Mala	Aceptable	Buena	Muy buena
Antes **de la pandemia**	☐	☐	☐	☐	☐
Ahora durante **la pandemia**	☐	☐	☐	☐	☐

37. Mi condición física cardio-respiratoria (capacidad para hacer ejercicio, por ejemplo, correr durante mucho tiempo) es:

	Muy mala	Mala	Aceptable	Buena	Muy buena
Antes **de la pandemia**	☐	☐	☐	☐	☐
Ahora durante **la pandemia**	☐	☐	☐	☐	☐

38. Mi fuerza muscular es:

	Muy mala	Mala	Aceptable	Buena	Muy buena
Antes **de la pandemia**	☐	☐	☐	☐	☐
Ahora durante **la pandemia**	☐	☐	☐	☐	☐

39.Mi velocidad/agilidad es:

	Muy mala	Mala	Aceptable	Buena	Muy buena
Antes **de la pandemia**	☐	☐	☐	☐	☐
Ahora durante **la pandemia**	☐	☐	☐	☐	☐

40. Mi flexibilidad es:

	Muy mala	Mala	Aceptable	Buena	Muy buena
Antes **de la pandemia**	☐	☐	☐	☐	☐
Ahora durante **la pandemia**	☐	☐	☐	☐	☐

COMPORTAMIENTO DE SUEÑO

41. ¿A qué hora sueles irte a la cama los días de semana (lunes a viernes, en formato 24 horas)?

a. Antes de la Pandemia

Hora	min	A.m/p.m

b. Ahora durante de la Pandemia

Hora	min	A.m/p.m

42. ¿A qué hora sueles irte a la cama los días de fin de semana (sábado y domingo)?

a. Antes de la Pandemia

Hora	min	A.m/p.m

b. Ahora durante de la Pandemia

Hora	min	A.m/p.m

43. ¿A qué hora sueles levantarte los días de semana (lunes a viernes)?

a. Antes de la Pandemia

Hora	min	A.m/p.m

b. Ahora durante de la Pandemia

Hora	min	A.m/p.m

44. ¿A qué hora sueles levantarte los días de fin de semana (sábado y domingo)?

a. Antes de la Pandemia

Hora	min	A.m/p.m

b. Ahora durante de la Pandemia

Hora	min	A.m/p.m

CUESTIONARIO DE HÁBITOS ALIMENTARIOS

Por favor responda las siguientes preguntas relacionadas con tus hábitos alimenticios.

45. ¿Desayunas habitualmente? Sí ☐ No ☐

46. Si tu respuesta fue sí en la pregunta anterior, describe brevemente qué desayunas habitualmente.

____

47. ¿Consumes alimentos no saludables? (Alto en calorías o azúcar o grasa, por ejemplo: papas fritas, chocolates, pasteles, etc.)

	Nunca	A veces	La mayor parte del tiempo	Siempre
Antes **de la pandemia**	☐	☐	☐	☐
Ahora durante **la pandemia**	☐	☐	☐	☐

48. ¿Con que frecuencia consideras que comes fuera de control?

	Nunca	A veces	La mayor parte del tiempo	Siempre
Antes **de la pandemia**	☐	☐	☐	☐
Ahora durante **la pandemia**	☐	☐	☐	☐

49. ¿Con que frecuencia comes alimentos fuera de tus comidas?

	Nunca	A veces	La mayor parte del tiempo	Siempre
Antes **de la pandemia**	☐	☐	☐	☐
Ahora durante **la pandemia**	☐	☐	☐	☐

50. ¿Cuántas comidas realizas en el día? (Desayuno, almuerzo, Once/cena, colación).

	1-2 comidas al día	3-4 comidas al día	5-6 comidas al día	Más de 6 comidas al día
Antes **de la pandemia**	☐	☐	☐	☐
Ahora durante **la pandemia**	☐	☐	☐	☐

51. ¿Tiene alguna sugerencia/observación sobre el cuestionario? ¡Cuéntenosla!

EL CUESTIONARIO HA TERMINADO. MUCHAS GRACIAS POR TU PARTICIPACIÓN, NOS AYUDARÁ A CONOCER MÁS SOBRE LA IMPORTANCIA DE LA ACTIVIDAD FÍSICA.
